# Insight into the Corrosion Inhibition Performance of *Pistia stratiotes* Leaf Extract as a Novel Eco-Friendly Corrosion Inhibitor for Mild Steel in 1 M HCl Solution

**DOI:** 10.3390/molecules29225243

**Published:** 2024-11-06

**Authors:** Andres Carmona-Hernandez, María Concepción Barreda-Serrano, Hugo Albeiro Saldarriaga-Noreña, Roy López-Sesenes, José Gonzalo González-Rodríguez, Edgar Mejía-Sánchez, Jorge Alberto Ramírez-Cano, Ricardo Orozco-Cruz, Ricardo Galván-Martínez

**Affiliations:** 1 Instituto de Ingeniería, Universidad Veracruzana, Boca del Río 94294, Mexico; andcarmona@uv.mx (A.C.-H.); zs22024500@estudiantes.uv.mx (M.C.B.-S.); jorgeramirez02@uv.mx (J.A.R.-C.); 2 Centro de Investigaciones Químicas-IICBA, Universidad Autónoma del Estado de Morelos, Cuernavaca 62209, Mexico; hsaldarriaga@uaem.mx; 3 Facultad de Ciencias Químicas e Ingeniería, Universidad Autónoma del Estado de Morelos, Cuernavaca 62209, Mexico; rlopez@uaem.mx; 4 Centro de Investigación en Ingeniería y Ciencias Aplicadas, Universidad Autónoma del Estado de Morelos, Cuernavaca 62209, Mexico; ggonzalez@uaem.mx; 5 Facultad de Ingeniería, Universidad Veracruzana, Orizaba 94452, Mexico; edmejia@uv.mx

**Keywords:** *Pistia stratoties*, mild steel, corrosion inhibitor, phytochemical compounds

## Abstract

In this research, the *Pistia stratiotes* leaf (PSL) extract was evaluated as a green corrosion inhibitor for the corrosion of mild steel in 1 M HCl using electrochemical measurements and surface characterization. Electrochemical impedance spectroscopy (EIS) spectra showed that the inhibitory activity of the phytochemical compounds enhanced with increasing concentration up to 400 ppm, which was reflected in the increase in the charge transfer resistance and double-layer capacitance. Regarding the effect of immersion time, EIS results indicated that the persistence of the PSL extract was between 4 h and 8 h of exposure time. From polarization curve (PCC) results, the best performance of the corrosion inhibitor was achieved at 400 ppm with an inhibition efficiency of 93.7%. The PSL extract acted as a mixed-type corrosion inhibitor. The adsorption of the phytomolecules on the metal surface obeyed the Langmuir isotherm through a mixed mechanism (physical and chemical interactions) dominated by physisorption. Scanning electron microscopy (SEM) examinations and energy-dispersive X-ray spectroscopy (EDS) elemental analysis of the corroded samples confirmed the anticorrosive protection of the PSL extract. Chemical characterization of the PSL extract by GC-MS revealed the presence of phytol, steroids, and aromatic and long-chain unsaturated fatty acid esters, in order of abundance. Chemical quantum calculations by DFT allowed for determining that the phthalic acid, di(2-propylpentyl) ester compound has the most significant potential to act as the main active component in corrosion inhibition activity.

## 1. Introduction

Corrosion is a natural process of the deterioration of metals and alloys caused by chemical and electrochemical reactions with the environment [1]. This phenomenon leads to the gradual degradation of mechanical properties and structural integrity of the metallic infrastructure. The costs produced by corrosion in a developed country are estimated to be between 3.5 and 5% of its gross domestic product (GDP) [2,3]. Mild steel is one of the most widely used materials in industry and construction applications due to its strength, durability, and low cost. However, this material has low corrosion resistance, especially in corrosive acidic media [4,5]. Mild steel is often exposed to acid solutions containing sulfuric or hydrochloric acid during the pickling process, which is a chemical cleaning process that serves to remove dirt and rust scales from the metal surface, with the risk of producing severe corrosion attack and promoting hydrogen embrittlement [6,7,8].

The use of corrosion inhibitors is one of the most effective corrosion prevention and control methods in acidic environments [9]. These are chemical substances that, when added in small concentrations to the corrosive medium, decrease the corrosion rate of the metal [10]. Corrosion inhibitors are widely used in various industries to protect metal equipment, structures, and components. However, many of the corrosion inhibitors used commercially are toxic and contaminate effluents, causing damage to human health and the environment [11,12]. For this reason, the scientific community, following the guidelines and directives of green chemistry, has focused on researching new ecological and sustainable alternatives for corrosion inhibitors that have low toxicity, are biodegradable, and provide good inhibition efficiency [13].

Natural extracts from plant species (leaves, peels, seeds, fruits, and roots) and organic matter waste have emerged as potential renewable and cheap sources of green corrosion inhibitors [14,15]. The natural extract is a complex mixture of phytochemical compounds whose chemical structure confers anti-inflammatory, antimicrobial, and antioxidant properties. Some natural products obtained through simple extraction methods are flavonoids, flavones, tannins, steroids, terpenes, and essential oils, among other active substances, which can participate synergistically in the corrosion inhibition process by adsorbing on the metal surface [16].

Medupin et al. [17] conducted an extensive review of plant extracts evaluated as corrosion inhibitors, as well as the several extraction methodologies and the effect of some factors, such as immersion time and temperature, on the inhibition performance. Finally, the authors emphasized the importance of comprehensively addressing corrosion in mild steel, considering the sustainability perspective that includes the economic, safety, and environmental impacts of inhibitors from plant extracts. Relevant recent studies have tested some plant extracts to inhibit the corrosion of mild steel or carbon steel in pickling media, such as *Persea americana* [18], *Acanthopanax senticosus* [19], *Calamintha* [20], *Cyclotrichium niveum* [21], *Ambrosia trifida* [22], *Artemisia absinthium* [23], *Ipomoea batatas* [24], *Platanus acerifolia* [25], and *Vateria indicia* [26], among others, obtaining encouraging results in which natural extracts have achieved corrosion inhibition efficiencies greater than 90% using concentrations between 100 and 1000 ppm. For example, Fouda et al. [27] evaluated the ethanolic *Verbena Officinalis* leaf extract as a corrosion inhibitor on carbon steel in 0.5 M H_2_SO_4_ solution. They reported an inhibition efficiency of 91% at 1000 ppm from electrochemical impedance spectroscopy (EIS) results. Likewise, Muhamma et al. [28] explored the methanolic *Kopsia teoi* extract as a corrosion inhibitor for mild steel in 0.5 M HCl, achieving an inhibition efficiency of 93.51% using electrochemical measurements.

*Pistia stratiotes*, commonly known as water lettuce, is a floating aquatic plant belonging to the Araceae family. It is native to tropical and subtropical regions but has become widely distributed worldwide due to its ability to thrive in various water conditions [29].

Water lettuce is considered an invasive plant due to its disproportionate growth and can cause serious ecological problems in water bodies, with economic and health repercussions for the population. Furthermore, the accumulation of this aquatic weed causes a reduction of dissolved oxygen and, consequently, the death of aquatic plants and fishes [30,31].

On the other hand, this plant has been shown to have antioxidant activity that could be beneficial for protecting metals against corrosion. For example, Herpandi et al. [32] extracted the active compounds from *Pistia stratiotes* using thin-layer and column chromatography. The authors reported high antioxidant activity and identified the presence of flavonoids and fatty acid compounds. Therefore, the main objective of this work was to explore *Pistia stratiotes* leaf (PSL) extract as a potential source of green corrosion inhibitor by using chemical characterization techniques for identifying bioactive compounds and studying the anticorrosive effect of PSL extract on mild steel in hydrochloric acid pickling medium using weight loss measurements and electrochemical and surface characterization techniques.

## 2. Results and Discussion

### 2.1. FT-IR and MS-GC Analysis

**Figure 1:**
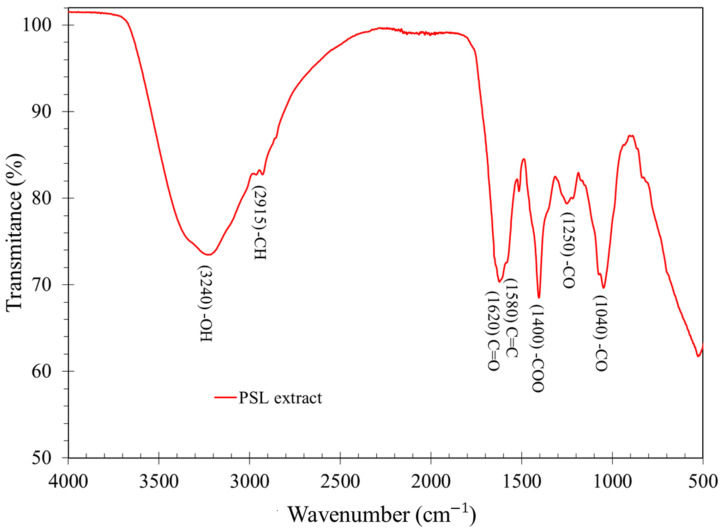
FTIR spectra of the PSL extract.

Figure 1 shows the FTIR spectra indicating the functional groups found in the PSL extract. The broad peak at 3240 cm^−1^ can be associated with the strong stretching vibrations of the hydroxyl (-OH) group. The peak at 2915 cm^−1^, overshadowed by the broad peak mentioned above, is related to the asymmetric stretching vibrations of -CH_2_ [33]. On the other hand, the overlapping peaks at 1620 and 1580 cm^−1^ can be attributed to the stretching of the carbonyl group (C=O) and the C=C double bond, respectively [1]. The sharp peak at 1400 cm^−1^ can be attributed to the presence of a COO– bond [34]. The peaks at 1250 and 1040 cm^−1^ correspond to the stretching vibrations of the C-O and C-O-C bonds, respectively [35]. As will be presented in the next section, compounds containing these functional groups were identified by GC-MS.

**Figure 2:**
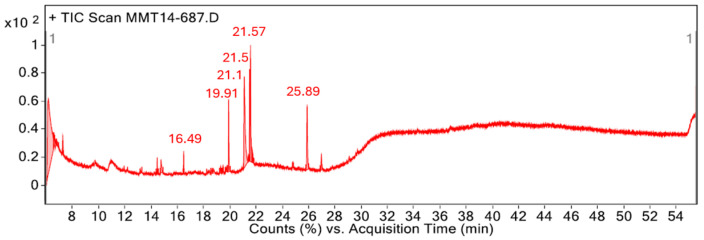
GC-MS chromatogram of PSL extract.

**Figure 3:**
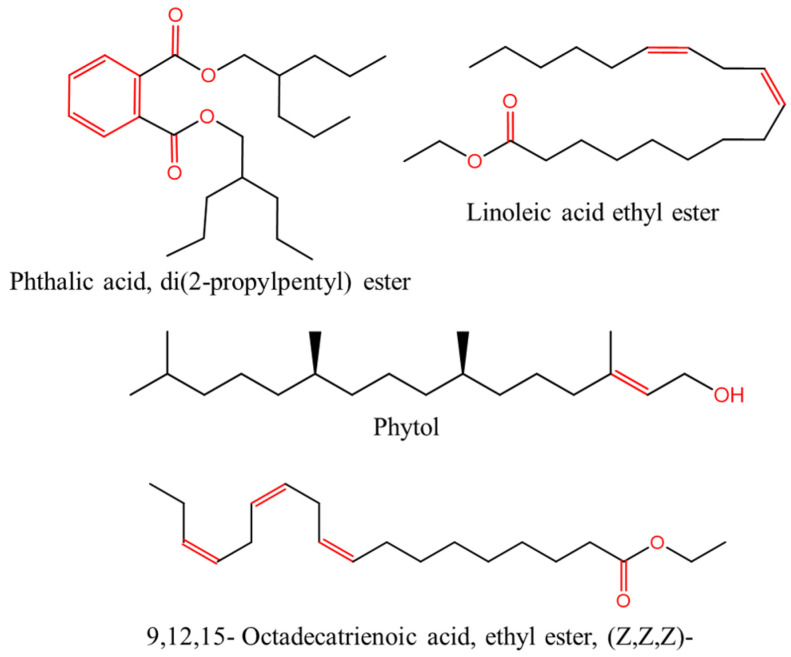
Chemical structures of the main constituents of the PSL extract.

The chemical composition of the PSL extract was characterized by GC-MS analysis. The GC chromatogram (Figure 2) and Table 1 show the major identified phytochemical compounds, their retention time (Rt), molecular weight, molecular formula, and peak area. Moreover, Table 1 includes the works in which the compounds have been identified from *Pistia stratiotes*. It is worth mentioning that 23 compounds were identified in total, summarized in Table S1 as supplementary material. Phytol was the compound with the highest percentage of peak area (33.59%). This diterpene is part of chlorophyll, which has exhibited antioxidant, antimicrobial, and anti-inflammatory activities [36]. The second compound in relative abundance, 9,12,15-Octadecatrienoic acid, ethyl ester, (Z,Z,Z)- (20.98%) is a steroid very similar to 9,12,15-Octadecatrienoic acid, methyl ester, (Z,Z,Z)-, which has been isolated from ethanolic PSL extract [37,38]. Ogunleye et al. [39] reported this compound as the major constituent in the Mangifera indica peel extract, which was also evaluated as a corrosion inhibitor in acidic medium. Phthalic acid, di(2-propylpentyl) ester (14.03%) is a type of bicyclic aromatic hydrocarbon ester with antimicrobial activity [40]. Although this compound has not been reported in *Pistia stratiotes* extracts, Ma et al. [41] found a compound with a similar chemical structure (Phthalic acid, cyclohexylmethyl propyl ester) in an ethyl acetate PSL extract. Finally, linoleic acid ethyl ester (11.15%) and (9.60%) hexadecanoic acid, ethyl ester are fatty acid esters, the latter being a compound with antioxidant activity [38]. As shown in Figure 3, the main phytochemical compounds identified by GC-MS analysis contain heteroatoms with unpaired electrons, unsaturated bonds, and aromatic rings, which act as active adsorption sites with the metal surface.

### 2.2. Open Circuit Potential (E_ocp_) Measurements

**Figure 4:**
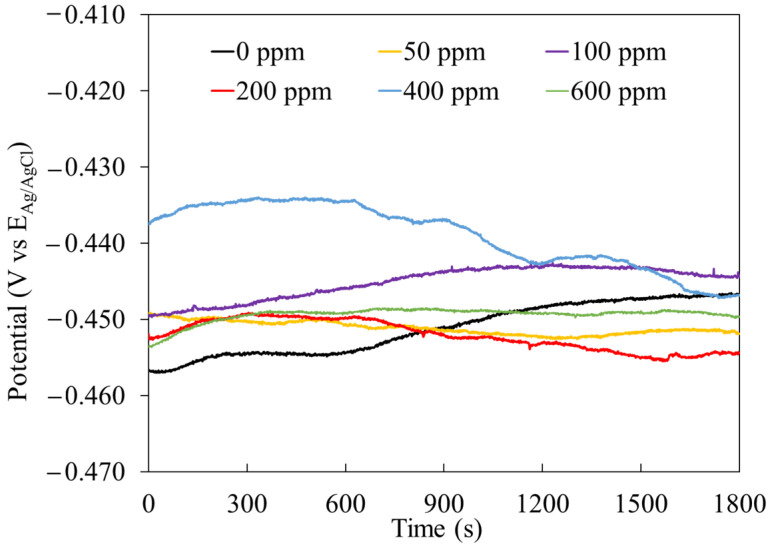
E_ocp_ measurement of mild steel in 1 M HCl solution at different concentrations of the PSL extract.

The E_ocp_ represents the potential value in which the anodic and cathodic reaction kinetics reach the stationary state. Figure 4 shows the E_ocp_ value of mild steel as a function of time in the acidic solution with different concentrations of the PSL extract. The shift in the E_ocp_ was a few mV compared with the E_ocp_ value in the absence of the corrosion inhibitor. This suggests that phytochemical molecules of the extract are adsorbed at both anodic and cathodic sites on the metal surface. The most electropositive values of E_ocp_ were obtained at 100 ppm, whereas the most electronegative values corresponded to the solution with 600 ppm of the inhibitor.

### 2.3. Electrochemical Impedance Spectroscopy (EIS)

**Figure 5:**
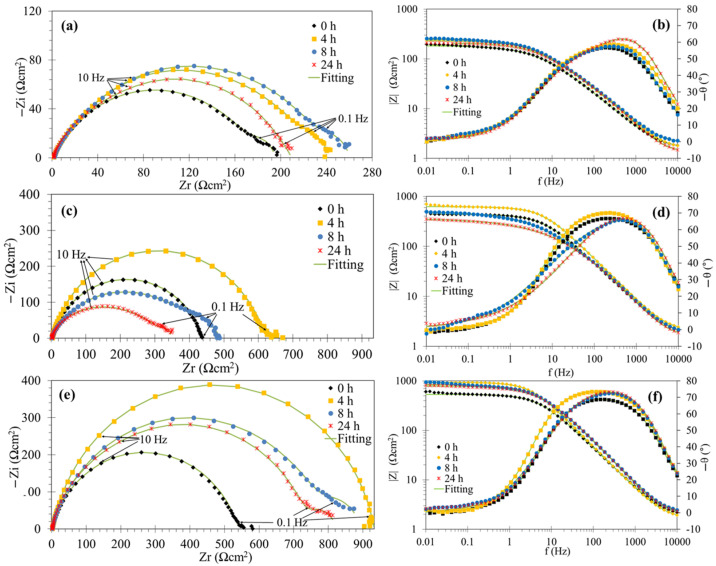
Nyquist (**left**) and Bode (**right**) plots of the mild steel in 1 M HCl solution in the absence (**a**,**b**) and with addition of 50 (**c**,**d**) and 100 ppm (**e**,**f**) of the PSL extract.

**Figure 6:**
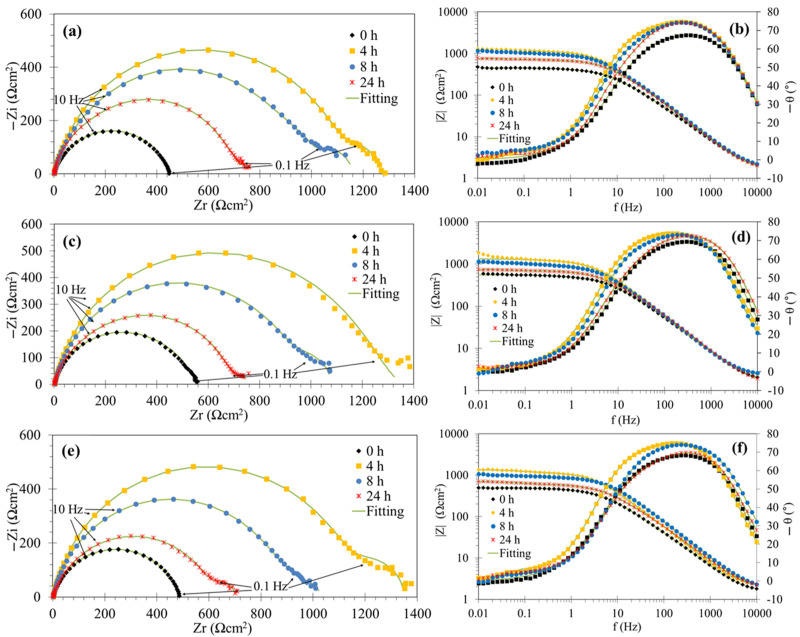
Nyquist (**left**) and Bode (**right**) plots of the mild steel in 1 M HCl solution with addition of 200 (**a**,**b**), 400 (**c**,**d**), and 600 ppm (**e**,**f**) of the PSL extract.

Figure 5 and Figure 6 show the EIS spectra for mild steel in the acidic solution at different immersion times without and with several concentrations of the PSL extract. In the absence of the corrosion inhibitor, the Nyquist curves (Figure 5a) showed apparently two depressed capacitive loops: the capacitive loop at high and intermediate frequencies was attributed to the charge transfer process in parallel with the effect of the electrochemical double-layer capacitance, and the smaller capacitive loop at lower frequencies can be associated with the adsorption of intermediate species during the anodic dissolution of Fe in acid solution in the presence of Cl^−^ ions [43,44]. In the inhibited system, the Nyquist curves (Figure 5a,c,e) also exhibited two semicircles with higher diameter in comparison to the blank solution, except at 0 h (for all concentrations) and 4 h (only at 100 ppm) of exposure, where a less depressed semicircle was apparently observed.

On the other hand, the time constants corresponding to the capacitive loops described in the Nyquist diagram were not distinguished in the Bode diagram of the phase angle (Figure 5b,d,f), which showed a unique broad peak both in the inhibited and uninhibited systems.

Regarding the effect of immersion time on the EIS spectra, the diameter of semicircles reached a maximum at 4 h of exposure and subsequently decreased at all concentrations of the PSL extract. This behavior can also be correlated with the magnitude of the impedance modulus (|Z|) at low frequencies as a function of time in the Bode plots. As the exposure time increased, the desorption of the PSL extract molecules occurred, and the intermediate complexes of the electrochemical reactions could be incorporated into the inhibitor film, deteriorating its anticorrosive properties. This was manifested in the appearance of the second semicircle at low frequencies after 4 h of exposure.

Finally, from Figure 6, it should be noted that the features and behavior of the EIS spectra as a function of time were very similar at concentrations greater than 100 ppm. This behavior suggests that the PSL extract can provide acceptable anticorrosive protection during the first hours of exposure to these inhibitor concentrations.

**Figure 7:**
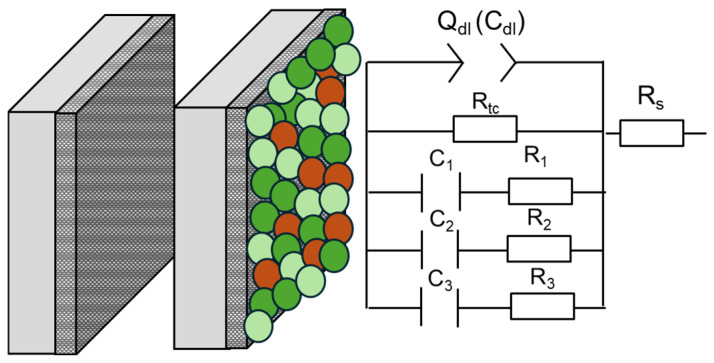
Equivalent circuit proposed for describing the metal–solution interface in the absence and presence of PSL extract.

To extract quantitative information from EIS spectra, several electrical equivalent circuits (EECs) were tested to fit the EIS data and model the metal–solution interface in the absence and presence of corrosion inhibitor film. The proposed EEC (Figure 7) is the electrical representation for the active dissolution mechanism of iron in acidic solutions containing chloride ions, which considers the adsorption of intermediate species [43,44]. With the addition of the PSL extract, competitive adsorption between these intermediate species and the phytochemical molecules can occur on the metal surface, surprising the anodic dissolution and hydrogen evolution reactions, as discussed in a later section. The simple Randles circuit was used to fit the EIS data of the inhibited system at 0 h of exposure. The fitted EIS spectra acquired with the ZSimpWin v. 3.22 software are shown in Figure 5 and Figure 6. Figure S1 shows examples of suitable fitting experimental EIS data using this software.

The electrical/electrochemical parameters included in the EEC are the solution resistance (R_s_), the charge transfer resistance (R_ct_), and the non-ideal capacitance of the electrochemical double layer represented by a constant phase element (Q_dl_). The non-ideal capacitive behavior in the electrochemical interface has been associated with the inhomogeneity of the metal surface, which produces a dispersion of the time constant. The impedance of Q (Z_Q_) is given by Equation (1) as follows:

(1) $$Z_{Q}=Y_{0}{\left(\omega j\right)}^{-n}$$

where Y_0_ and n are the admittance and exponential parameters of the constant phase element, respectively. The effective double-layer capacitance (C_dl_) can be calculated from the parameters of Q by applying Brug’s equation [45]:

(2) $$C_{dl}={Y_{0}}^{\frac{1}{n}}{\left({R_{s}}^{-1}+{R_{ct}}^{-1}\right)}^{\frac{n-1}{n}}$$

The remaining electrical parameters C_1_, R_1_, C_2_, R_2_, C_3_, and R_3_ involve the adsorption of the intermediate species generated during the anodic dissolution in acid solution in the presence of chloride ions. The R_1_-C_1_, R_2_-C_2_, and R_3_-C_3_ elements in parallel are related to the coverages and adsorption kinetics of (FeOH)_ads_, (FeCl)^−^_ads_, and (FeCl)_ads_. Since the anodic dissolution of Fe depends on the adsorption of these intermediate species, the adsorption of the inhibitor molecules is limited to the active sites where the adsorption of these intermediate species could take place. Table 2 summarizes the electrochemical parameters obtained by fitting using the EEC of Figure 7. The inhibition efficiency from the EIS results is calculated according to Equation (3):

(3) $$\eta =\frac{R_{ct(inh)}-R_{ct(0)}}{R_{ct(inh)}}*100$$

where R_ct(0)_ and R_ct(inh)_ are the charge transfer resistance in the absence and presence of PSL extract, respectively, and η is the corrosion inhibition efficiency. As seen in Table 2, the R_ct_ value, the main parameter related to the kinetics of electrochemical reactions, increased with the addition of PSL extract, producing an increase in the R_ct_ value of up to an order of magnitude at concentrations above 100 ppm. Furthermore, the inhibition effect improved as the extract concentration increased, reaching maximum inhibition efficiency (82.69%) at 400 ppm at 4 h of exposure. The further addition of PSL extract (600 ppm) did not significantly change the inhibition efficiency. This fact reflects that a greater amount of phytomolecules can be adsorbed on the active sites of the metal surface, hindering the charge transfer process up to a critical concentration. In contrast, the C_dl_ values decreased in the presence of PSL extract, showing a strong decrease in this parameter at concentrations greater than 200 ppm. According to the Helmholtz model, which assumes the behavior of the electrochemical double layer as a parallel plate capacitor, the decrease in the C_dl_ value can be caused by increasing the thickness of the double layer and/or by decreasing the local dielectric constant at the interface because the adsorption of larger phytomolecules replaces the previously adsorbed water molecules (modification of the dielectric material) on the metal surface.

On the other hand, the parameter n increased in the inhibited solution, which can be associated with the formation of a compact and homogeneous inhibitor film that reduced corrosion attack and the roughness of the metal surface [46].

### 2.4. Potentiodynamic Polarization Curves (PPCs)

**Figure 8:**
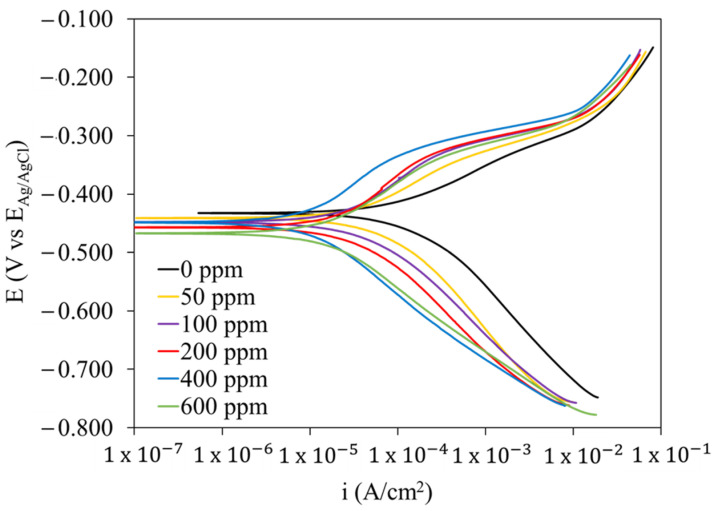
PPC of mild steel in 1 M HCl solution at different concentrations of the PSL extract after 4 h of exposure.

Polarization curves of mild steel in the acid solution at different concentrations of the PSL extract at 4 h of exposure time are shown in Figure 8, and their electrochemical parameters arising from the Tafel extrapolation method are summarized in Table 3, including the corrosion potential (E_corr_), anodic and cathodic Tafel slopes (b_a_ y b_c_), and the corrosion current density (i_corr_). As shown in Figure 8, the presence of PSL extract shifted the anodic and cathodic branches towards lower current density values, slightly modifying the anodic and cathodic slopes. As shown in Table 3, the general trend of the Tafel slope values was that the b_a_ values increased and the b_c_ values decreased as more PSL extract was added. Moreover, the E_corr_ value shifted up to 32 mV in the cathodic direction. Based on the criterion of the E_corr_ shift of 85 mV to classify a chemical compound in a corrosion inhibitor type, as well as the effect of the addition of the corrosion inhibitor in the Tafel slopes, these results suggest that the phytochemical compounds in the PSL extract acted as mixed-type corrosion inhibitors, suppressing both the anodic and cathodic reactions and with a predominant effect on the cathodic reactions [47,48].

The corrosion inhibition performance in terms of i_corr_ values was calculated according to Equation (4) as follows:

(4) $$\eta =\frac{i_{corr(0)}-i_{corr(inh)}}{i_{corr(0)}}*100$$

Table 3 shows that η values increased as the concentration of the PSL extract increased until reaching a maximum η value at 400 ppm. Nevertheless, a further addition of corrosion inhibitor at 600 ppm led to a slight decrease in the inhibition efficiency. This can be attributed to the competitive adsorption of the inhibitor molecules on the metal surface, the repulsion forces between them becoming significant and causing their desorption from the metal surface once a critical concentration is exceeded [49].

### 2.5. Surface Analysis by SEM and EDS

**Figure 9:**
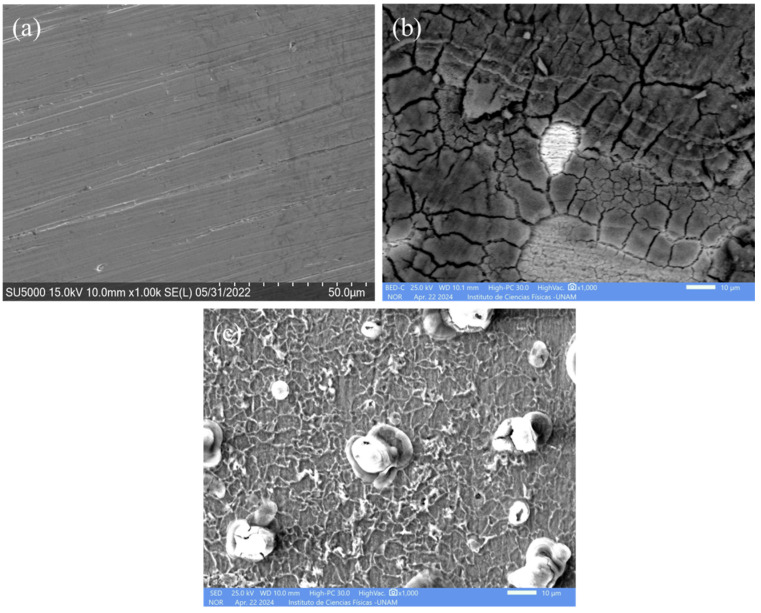
SEM images of the mild steel surface (**a**) before immersion and after 24 h of exposure in the 1 M HCl solution (**b**) without and (**c**) with 400 ppm addition of PSL extract.

Figure 9 shows the SEM micrographs of the mild steel surface before immersion and after 24 h of exposure to the uninhibited and inhibited solution. Before immersion (Figure 9a), a clean surface, free of rust and polishing marks, is expected. As shown in Figure 9b, the surface exposed in the absence of inhibitor exhibited a general corrosion attack with the formation of porous and cracked corrosion products. In contrast, the steel surface showed a lower degree of corrosion attack, as it was covered with a thin protective layer. Even marks produced by polishing can still be seen. The EDS analysis of the corroded surfaces confirmed the formation of a protective corrosion inhibitor film, as shown in Table 4. It can be noted the considerable decrease in the content of Fe (from 95.52% to 60.98%), the increase in the content of O (from 0.23% to 29.65%), and the presence of Cl^−^ (4.62%) on the corroded surface in the aggressive solution in comparison with the reference surface. It is indicative that the steel underwent a severe corrosion attack. Instead, the addition of 400 ppm of the PSL extract led to an increase in the content of Fe (85.16%) and a decrease in the content of Cl^−^ (0.19%) and O (8.90%) with respect to the blank solution, which can be attributed to the adsorption of the organic molecules present in the PSL extract and the anticorrosive properties of the corrosion inhibitor film.

### 2.6. Adsorption Isotherm

Generally, organic compounds inhibit the corrosion of steel through an adsorption process on the metal surface, which can be described by an adsorption isotherm. In order to extract basic thermodynamic information on the nature of the interaction between the inhibitor molecules and the metal surface, the experimental data of the inhibition efficiencies derived from the electrochemical techniques were fitted with the linearized expressions of Langmuir (5), Temkin (6), Freundlich (7), and Frumkin (8) isotherms as follows [50]:

(5) $$\frac{C_{inh}}{\theta }=\frac{1}{K_{ads}}+C_{inh}$$

(6) $$\theta =\frac{1}{f}\ln K_{ads}+\frac{1}{f}\ln C_{inh}$$

(7) $$\ln\theta =\frac{1}{n}\ln C_{inh}+\ln K_{ads}$$

(8) $$\ln\frac{\theta }{(1-\theta )C_{inh}}=\ln K_{ads}+f\theta$$

Ideally, the surface coverage (θ) is directly related to inhibition efficiency (θ = η/100), and K_ads_ is the adsorption equilibrium constant. In the Temkin and Frumkin model, *f* represents the attraction/repulsion interaction between organic molecules. In the Freundlich model, the parameter n considers the effects of surface heterogeneity and interaction forces between molecules [51].

**Figure 10:**
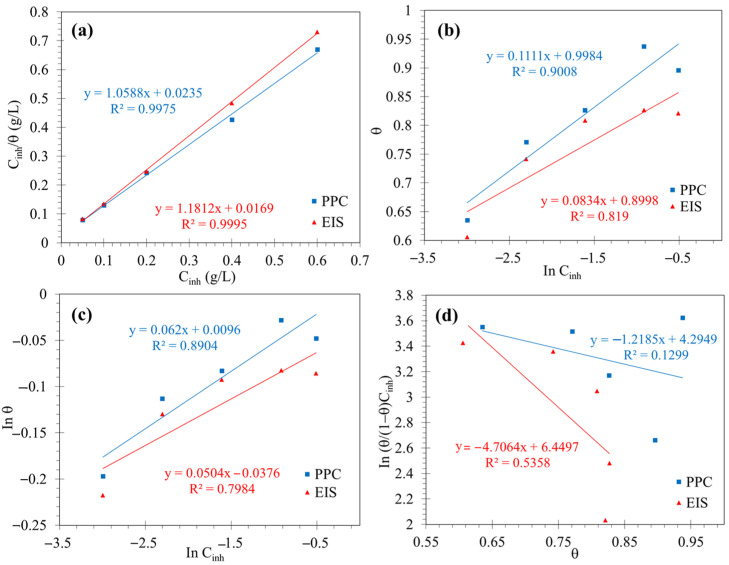
Adsorption isotherms plotted from electrochemical measurements: (**a**) Langmuir, (**b**) Temkin, (**c**) Freundlich, and (**d**) Frumkin.

Figure 10 illustrates the linear regression of the experimental data with each of the adsorption isotherms, finding that the model that presented a better fit was the Langmuir model (the linear regression coefficient R^2^ was close to unity). This model assumes the formation of a monolayer, and there is no interaction between the adsorbed molecules. Nevertheless, the slight deviation from the slopes calculated with the unit (Figure 10a) has generally been attributed to orientation, conglomeration, and interaction forces between the adsorbed organic molecules and variation of the adsorption energies as the θ increases [44]. Therefore, the adsorption of phytochemical compounds of the PSL extract can be described according to the Langmuir isotherm without neglecting the interactions between the molecules.

Table 5 shows the thermodynamic parameters of adsorption derived from the Langmuir model. The K_ads_ (in L/g) values were determined graphically by the intersection of the straight line with the y-axis in the C_inh_/θ vs. C_inh_ plot (Figure 10a), and the Gibbs free energy (∆G_ads_, in kJ/mol) was computed according to the following equation:

(9) $${∆G}_{ads}=-RT\ln(C_{solv}K_{ads})$$

where R is the universal gas constant (8.314 J/mol. K), T is the absolute temperature (K), and C_solv_ is the concentration of water (1000 g/L). The magnitude of ∆G_ads_ has been used to predict the type of adsorption mechanism of the inhibitor molecules. ∆G_ads_ values close to or above −20 kJ/mol are related to physisorption, whereas ∆G_ads_ values in the order of −40 kJ/mol indicate chemisorption [50]. As shown in Table 5, the ∆G_ads_ calculated from the experimental data were in the range of −26 to −28 kJ/mol, suggesting that the compounds with corrosion inhibition activity contained in the PSL extract were adsorbed through a combination of physisorption and chemisorption (mixed adsorption), with a predominance of physisorption [52]. The chemical structure of the phytochemical compounds presents functional groups that can interact with the surface through covalent chemical bonds (chemisorption). At the same time, electrostatic interactions between the molecules and the metal surface can occur simultaneously. The mechanism of inhibition of the PSL extract on the corrosion of mild steel in the acidic solution will be discussed in more detail in a later section.

### 2.7. Quantum Chemical Calculations

Quantum chemical calculations using DFT provide a powerful tool to study the electronic structure of molecules and correlate it with their performance as corrosion inhibitors. The ground-state optimized geometries of the main compounds of the PSL extract, along with their HOMO, LUMO, and electrostatic potential (ESP) regions, are shown in Table 6. The HOMO orbital represents the regions of the molecule capable of donating electrons to the unoccupied d orbitals of the metal, whereas the LUMO orbital involves regions with the ability to accept electrons from the metal. From the ESP distribution, the positive (blue) and negative (red) regions indicate the zones with electrophilic and nucleophilic activity, respectively. For phytol, the electron density of the HOMO and LUMO orbitals comprises the OH hydroxyl group and the C=C double bond and extends to the five C atoms next to the OH group. Steroid and linoleic acid ethyl ester are compounds with similar chemical structure, so they share similar regions of the HOMO and LUMO orbitals distributed in the C=C double bonds and the -COO- ester group, respectively. Finally, the frontier orbitals of the phthalate ester molecule are mainly distributed in the benzenedicarboxylate moiety. These regions identified in the phytochemical compounds represent the active sites where the molecule can adsorb onto the metal surface.

The quantum chemical descriptors of the phytomolecules present in the PSL extract are summarized in Table 7. Regarding frontier orbital energy values, a high E_HOMO_ value has been related to the electron-donating property of a compound, whereas a low E_LUMO_ value has been associated with its electron-withdrawing capacity [53]. According to Table 7, the steroid compound presented the highest value of E_HOMO_, suggesting that this molecule more easily donates electrons to the metal surface. The molecule of phthalic acid, di(2-propylpentyl) ester, had the lowest value of E_LUMO_, indicating that this molecule has a greater capacity to capture electrons from the metal surface. Likewise, the ΔE_gap_ is closely related to the reactive capacity of the molecule. Low values of ΔE_gap_ represent that a lower amount of energy is required to remove an electron from the HOMO orbital to be transferred to the unoccupied orbitals on the metal surface [54]. According to the descending order of the ΔE_gap_ value, the phytochemical compounds followed the order linoleic acid ethyl ester > phytol > 9,12,15-Octadecatrienoic acid, ethyl ester, (Z,Z,Z)- > phthalic acid, di(2-propylpentyl) ester. Previous studies [55,56] have already reported values of ΔE_gap_ for linoleic acid ethyl ester and phytol of 6.78 and 6.88 eV, respectively, consistent with those obtained in this theoretical study.

On the other hand, phthalic acid, di(2-propylpentyl) ester had the lowest value of η_Q_ and highest value of χ, which suggests that this molecule offers less resistance to the charge transfer process and has a greater capacity to attract electrons from the metal, respectively. Hence, phthalic acid, di(2-propylpentyl) ester had the best electronic and structural properties for acting as a corrosion inhibitor compared to the other phytomolecules. Finally, the parameter ΔN determines the direction of electron transfer in the interaction between the metal and the inhibitor molecule. If ΔN < 0, electrons are transferred from the metal surface to the inhibitor molecule, whereas if ΔN > 0, electrons are transferred from the inhibitor molecule to the metal surface. All phytochemical compounds had positive ΔN values. This indicated that phytomolecules transfer electrons to the metal surface.

#### Local Reactivity Fukui Functions

**Figure 11:**
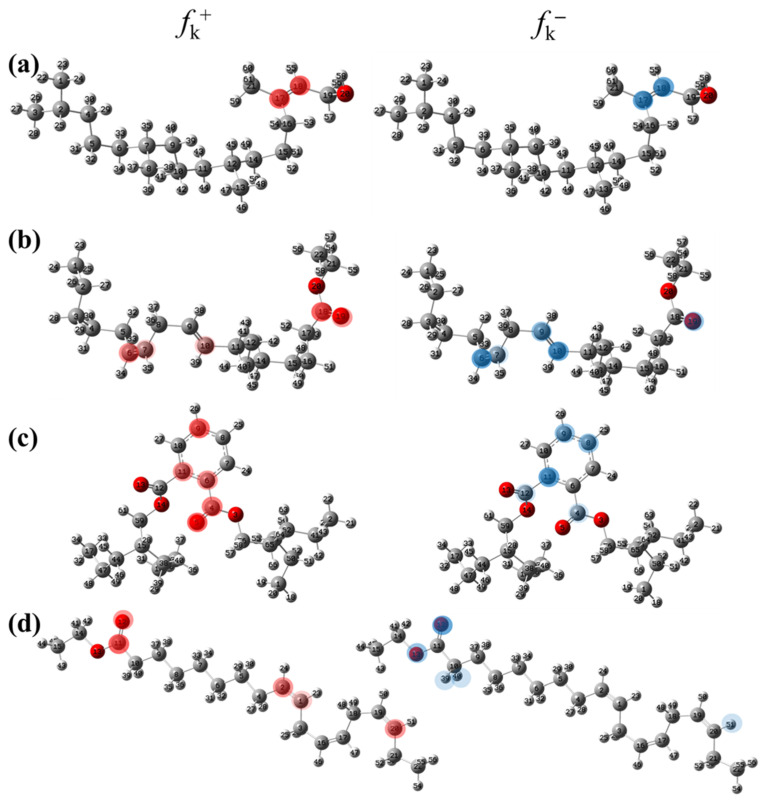
Most reactive atoms of the main compounds of the PSL extract based on the condensed Fukui functions: (**a**) phytol, (**b**) 9,12,15-Octadecatrienoic acid, ethyl ester, (Z,Z,Z)-, (**c**) phthalic acid, di(2-propylpentyl) ester, (**d**) linoleic acid ethyl ester.

The condensed Fukui functions are descriptors of the local reactivity of a molecule and allow the prediction of atoms susceptible to nucleophilic (f_k_^+^) and electrophilic attack (f_k_^−^). These descriptors were calculated using the following equations [57]:

(10) $$f_{k}^{+}=q_{k(N+1)}-q_{k\left(N\right)}$$

(11) $$f_{k}^{-}=q_{k(N)}-q_{k\left(N-1\right)}$$

where q_k(N)_, q_k(N+1)_ and q_k(N−1)_ are the charge values of k atom for neutral, anionic, and cationic forms of the molecule, respectively. The higher the value of f_k_^+^ or f_k_^−^, the greater the nucleophilic (ability to accept electrons) or electrophilic character (ability to donate electrons) of the atom, respectively. Figure 11 illustrates the most reactive atoms in the main phytochemical compounds in the PSL extract, and the Fukui function values are listed in Table S2. As shown in Figure 11a, the double bond (C17 and C18 atoms) is the main site involved in electrophilic and nucleophilic interactions for phytol. In the case of the steroid compound (Figure 11b), the atoms in the carbonyl group (C11, O12) and double bonds (C2, C1, and C20, C19) were estimated as the most active center for nucleophilic attack, whereas the O12, O13, H39, H40, and H51 atoms presented the most electrophilic character. For the phthalic ester compound (Figure 11c), the most reactive atoms are distributed in the aromatic ring (C9, C11, C6, C8) and the esters group (C4, O5, C12). Finally, in linoleic acid ethyl ester (Figure 11d), the most susceptible atoms to nucleophilic attack were O19 and C18 from the carbonyl group, and for electrophilic interactions, they were the C9, C10, and C6 from the double bonds.

In summary, electrophilic sites, such as O atoms and C=C double bonds, contain unpaired electron pairs and delocalized π electrons, respectively, which can be donated to the metal surface, forming coordinated covalent bonds with the unoccupied orbitals of the metal atoms. Double bonds of aromatic rings can also act as nucleophilic sites, accepting electrons from the metal through the process of back donation. These predictions are in agreement with the global reactivity observed in the ESP distribution and the HOMO and LUMO regions.

### 2.8. Corrosion Inhibition Mechanism by PSL Extract

The corrosion inhibition of mild steel in acidic solution by organic compounds occurs through the adsorption of these molecules, blocking the anodic and cathodic sites and, in turn, forming a protective layer that isolates the metal surface from the electrolytic medium. In the absence of PSL extract, the electrochemical reactions that take place are as follows [58,59]:

Anodic sites (iron dissolution):

(12) $$Fe+H_{2}O+{Cl}^{-}↔{\left[FeClOH\right]}_{ads}^{-}+H^{+}+e^{-}$$

(13) $${\left[FeClOH\right]}_{ads}^{-}↔{\left[FeClOH\right]}_{ads}+e^{-}$$

(14) $${\left[FeClOH\right]}_{ads}+H^{+}\to {Fe}^{2+}+{Cl}^{-}+H_{2}O$$

Cathodic sites (hydrogen evolution):

(15) $$Fe+H^{+}\to {\left(FeH^{+}\right)}_{ads}$$

(16) $${\left(FeH^{+}\right)}_{ads}+e^{-}\to {\left(FeH\right)}_{ads}$$

(17) $${\left(FeH\right)}_{ads}+H^{+}+e^{-}\to Fe+H_{2}$$

**Figure 12:**
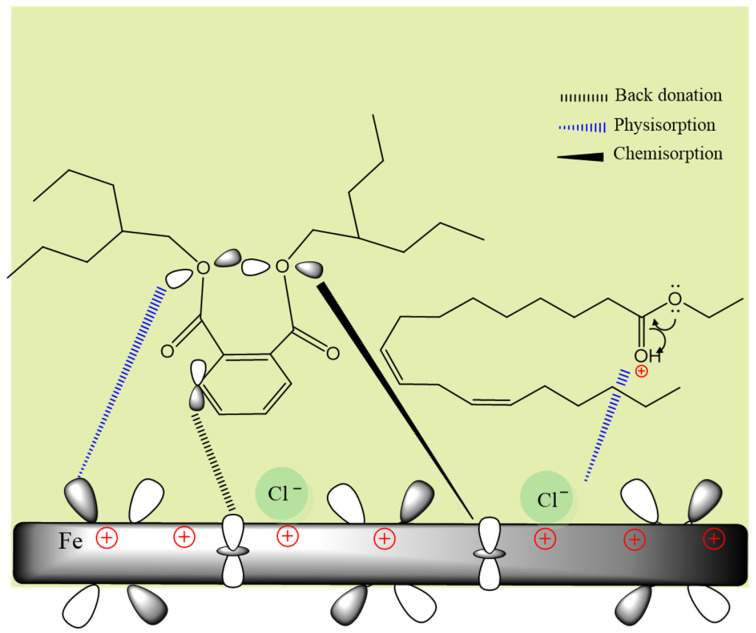
Schematic illustration of the adsorption mechanism of main compounds of the PSL extract on mild steel surface.

It is worth noting that the dissolution of Fe can occur simultaneously without the participation of the Cl^−^ ion via the adsorbed intermediate [FeOH]_ads_ [59]. In the presence of the PSL extract, there is a competitive adsorption between the intermediate complexes from electrochemical reactions and the phytochemical compounds. Figure 12 schematizes some of the physical and chemical interactions of adsorption of some phytochemical compounds of the PSL extract on the metal surface. By determining the zero-charge potential, it has been confirmed that the metal surface is positively charged [60]. This causes the adsorption of chloride ions to the metal by electrostatic attraction, which, in turn, produces electrostatic interactions (physisorption) with the partially positively charged sites of the inhibitor molecules because some of the phytomolecules present in the PSL extract can become protonated in the nucleophilic sites in acid solution. The O atom of the carbonyl group can be protonated and in equilibrium with its neutral form because the adsorbed protonated molecules can compete with H^+^ ions for electrons on the metal surface. This results in the release of H_2_ gas and the return of the carbonyl group to its neutral form [61].

On the other hand, chemical interactions between phytomolecules and metal surfaces can take place in electrophilic sites, i.e., unpaired electrons of O atoms can be donated to the unoccupied d orbitals of Fe atoms via coordinate covalent bonds. Likewise, the delocalized π electrons of aromatic rings and double bonds (HOMO region) can be transferred to the Fe atoms, and vice versa, d orbitals of the Fe atoms donate electrons to the antibonding π* orbitals (LUMO) of the inhibitor molecules through the back donation process [62].

### 2.9. Comparison of the PSL Extract with Other Plant-Based Corrosion Inhibitors

The investigation of natural plant-based extracts as sources of green corrosion inhibitors has been one of the main strategies for obtaining non-toxic, biodegradable compounds with anticorrosive properties at a low cost. According to the review of recent research on the evaluation of green corrosion inhibitors from plant extracts for mild steel corrosion in HCl (Table 8), the corrosion inhibition efficiency reported in these studies ranges from 87 to 96% using concentrations between 200 and 1500 ppm. Based on the findings of the present study, the corrosion inhibition performance of the PSL extract was comparable with other plant extracts reported previously. Therefore, the *Pistia stratoties* plant can be considered a potential source of green and environmentally friendly corrosion inhibitors.

## 3. Materials and Methods

### 3.1. Material and Test Solution

The test material was mild steel plates with chemical composition (in %wt) of 0.25% C, 0.28% Si, 0.2% Cu, 0.1% Mn, 0.05% S, 0.04% P, and the remainder Fe. Mild steel specimens of 20 × 20 × 5mm were machined for weight loss measurements, whereas cubic samples of 10 × 10 × 10 mm embedded in epoxy resin with a working area of 1 cm^2^ were made for electrochemical measurements. The test electrolyte was 1 M HCl pickling solution, prepared using 36.5% analytical grade HCl and distilled water.

### 3.2. Preparation of the Pistia Stratiotes Leaves (PSL) Extract

The collection of PSL was carried out in a lagoon located in Veracruz, Mexico. After the collection, the leaves were cleaned and dried in an oven at 45 °C for 24 h to remove moisture. After that, the dried leaves were ground to obtain a fine powder. The extraction by maceration was conducted by adding 50 g of the powder in an ethanol–water solution (70% *v*/*v* ethanol concentration) in a mass ratio of 1:20. The solution was stirred at 120 rpm for 72 h at room temperature. Finally, the solution was filtered with filter paper and evaporated using a rotary vacuum evaporator at 50 °C to eliminate the solvent. The extraction yield was 12%. The solid resulting from the evaporation of the solvent was weighed in specific quantities and dissolved in a 100 mL volumetric flask (volume of the test solution) to reach the corresponding concentrations of 50, 100, 200, 40, and 600 ppm.

### 3.3. Chemical Characterization of PSL Extract

To identify the functional groups contained in the PSL extract, Fourier Transform Infrared (FT-IR) spectroscopic analysis was conducted using a Nicolet 6700 spectrometer (Thermo Scientific, Waltham, MA, USA) in the wavelength range of 500–4000 cm^−1^.

The gas chromatography–mass spectrometry (GC-MS) analysis was performed by an 7890B GS/MS Triple Quad gas chromatograph (Agilent Technologies Co., Ltd., Santa Clara, CA, USA) associated with a fused HP 5MS capillary column (Agilent Technologies Co., Ltd., Santa Clara, CA, USA) and coupled with a 7999D mass spectrometer detector (Waters Corporation, Milford, MA, USA), which was employed to identify the phytochemical compounds present in the PSL extract. Dichloromethane was used as the carrier gas with a flow rate of 10 mL/min, and 4 µL of the sample was injected. Regarding the oven temperature program, it started at 40 °C for 1 min and increased to 286 °C by a heating ramp rate of 10 °C/min, and then the temperature was set at 285 °C for 25 min. For MS detection, an ionization voltage of 70 eV with 150 °C for the ion source temperature and a mass range of 20 to 600 *m*/*z* were used to acquire the electron ionization MS.

### 3.4. Electrochemical Characterization

Electrochemical measurements were performed using an SP150 BioLogic electrochemical workstation (BioLogic SAS, Clais, France). The experimental setup consisted of a conventional three-electrode arrangement in a glass cell containing 200 mL of the test solution. The coupons of mild steel were the working electrodes (WEs), the Ag/AgCl (sat., KCl) was used as the reference electrode (RE), and a graphite rod acted as a counter electrode (CE). Before each electrochemical test, the open circuit potential (E_ocp_) of the WEs was measured for 30 min or until a stationary value was reached.

For EIS measurements, a sinusoidal signal of 10 mV vs. E_ocp_ of amplitude was applied in the frequency range of 10,000 Hz to 10 mHz, with 10 points per frequency decade. PPC measurements were conducted with an overpotential of ±250 mV vs. E_ocp_ using a scan rate of 1 mV/s. All electrochemical tests were carried out at atmospheric pressure and room temperature in Veracruz Port, México.

### 3.5. Surface Characterization by SEM and EDS

Surface analysis of the corroded surfaces was carried out using a scanning electron microscope (SEM) JEOL JSM-IT500 (JEOL Ltd., Tokyo, Japan) equipped with an energy-dispersive X-ray spectrometer (EDS) Bruker XFlash 6|30 (Bruker Nano GmbH, Berlin, Germany). The steel samples were exposed to the acid solution with and without the PSL extract for 24 h.

### 3.6. Density-Functional Theory (DFT) Calculations

Quantum chemical calculations were carried out to compute the electronic properties and chemical reactivity of the main phytochemical compounds identified in the PSL extract. All calculations were implemented using the Gaussian 09 software package. The optimized structure of these compounds was calculated by DFT using the Lee–Yang–Parr correlation function (B3LYP) at the level of theory 6–31G (d, p) basis set [66]. The main quantum chemical parameters involved in the corrosion inhibition performance of a compound, such as the energy gap (ΔE_gap_), ionization potential (I), electron affinity (A), global hardness (η), electronegativity (χ), and fraction of transferred electrons (ΔN), were calculated from the highest occupied molecular orbital (E_HOMO_) and lowest unoccupied molecular orbital energy (E_LUMO_) values according to the following equations:

$${∆E}_{Gap}=E_{HOMO}-E_{LUMO}$$

$$I_{p}=-E_{HOMO}$$

$$A_{e}=-E_{LUMO}$$

$$\chi =\frac{I_{p}+A_{e}}{2}$$

$$\eta =\frac{I_{p}-A_{e}}{2}$$

$$\Delta N=\frac{\chi_{Fe}-\chi_{inh}}{2(\eta_{Fe}+\eta_{inh})}$$

where χ_Fe_ and η_Fe_ are the electronegativity and hardness of iron, with their theoretical values being 0 and 7 eV/mol, respectively [67].

Finally, the nucleophilic and electrophilic sites of the molecules present in the PSL extract were estimated by calculating condensed Fukui functions using the UCA-FUKUI v. 2.1 software via the Finite Difference (FD) and natural population analysis [68].

## 4. Conclusions

The present work evaluated the effect of PSL extract on the corrosion inhibition of a mild steel 1 M HCl solution. The main conclusions drawn from the theoretical and experimental results were the following:

- Electrochemical measurements results indicated that the inhibition efficiency of the PSL extract enhanced as concentration increased until reaching a maximum efficiency of 87–91% at 400 ppm. Further addition of the PSL extract produced a slight decrease in the protective properties of the inhibitor film against corrosion.
- Polarization curve results revealed that the PSL extract acted as a mixed-type corrosion inhibitor, adsorbing at both the anodic and cathodic sites without strongly modifying the mechanism of electrochemical reactions.
- Adsorption isotherms analysis indicated that the adsorption of the phytochemical molecules present in the extract can be governed by the Langmuir isotherm through a mixed adsorption process, with the physisorption process dominating.
- Surface characterization by SEM and EDS showed that the compounds present in the PSL extract were adsorbed on the metal surface, reducing corrosion attack.
- Chemical characterization by GC-MS and FTIR allowed the identification of phytochemical compounds such as phytol, steroids, unsaturated long-chain aliphatic esters, and aromatic esters in the PSL extract.
- Quantum chemical calculations by DFT determined that the phthalic acid, di(2-propylpentyl) ester has the chemical structure with the best electronic properties and active sites with which it can interact with the metal surface compared to the other components identified in the extract.

## Figures and Tables

**Table 1:** Main constituents identified in the PSL extract by GC-MS analysis.

Compound	Rt (min)	Molecular Weight	Molecular Formula	Area (%)	Ref.
Phytol	21.1	296	C_20_H_40_O	33.59	[42]
9,12,15-Octadecatrienoic acid, ethyl ester, (Z,Z,Z)-	21.57	306	C_20_H_34_O_2_	20.98	
Phthalic acid, di(2-propylpentyl) ester	25.89	390	C_24_H_38_O_4_	14.03	
Linoleic acid ethyl ester	21.50	308	C_20_H_36_O_2_	11.15	
Hexadecanoic acid, ethyl ester	19.91	284	C_18_H_36_O_2_	9.60	[42]

**Table 2:** Electrochemical parameters derived from the fitting of EIS spectra using the proposed electrical equivalent circuits.

C_inh_ (ppm)	t (h)	R_s_ (Ωcm^2^)	Q_dl_.Y_0_ (Ss^n^/cm^2^)	n	R_ct_ (Ωcm^2^)	C_1_ (F/cm^2^)	R_1_ (Ωcm^2^)	C_2_ (F/cm^2^)	R_2_ (Ωcm^2^)	C_3_ (F/cm^2^)	R_3_ (Ωcm^2^)	C_dl_ (μF/cm^2^)	χ^2^ × 10^4^	η (%)
0	0	1.54	5.82 × 10^−4^	0.71	194	4.92 × 10^−6^	774.70	8.13 × 10^−4^	2277	1.41 × 10^−5^	0.20	32.94	0.71	
	4	1.38	4.43 × 10^−4^	0.72	241	8.59 × 10^−6^	521.90	3.31 × 10^−4^	3861	1.28 × 10^−5^	0.50	23.48	0.38	
	8	1.69	4.25 × 10^−4^	0.71	259	8.44 × 10^−6^	648.60	5.84 × 10^−4^	3759	1.26 × 10^−5^	0.93	23.55	1.07	
	24	1.26	3.82 × 10^−4^	0.72	208	5.68 × 10^−6^	283.50	2.06 × 10^−5^	699	2.10 × 10^−5^	0.04	20.71	1.22	
50	0	1.55	1.09 × 10^−4^	0.82	434							16.24	0.90	55.33
	4	1.94	7.16 × 10^−5^	0.85	614							14.93	0.62	60.59
	8	1.85	2.80 × 10^−4^	0.72	487	4.38 × 10^−6^	1217	1.28 × 10^−4^	4804	1.47 × 10^−5^	0.02	14.76	0.84	46.81
	24	1.59	2.96 × 10^−4^	0.72	348	2.76 × 10^−6^	27	5.61 × 10^−4^	5656	1.37 × 10^−5^	1.07	15.06	0.98	40.03
100	0	1.75	9.95 × 10^−5^	0.83	532							16.89	0.64	63.53
	4	1.59	7.12 × 10^−5^	0.86	936							16.22	0.86	74.18
	8	2.16	7.28 × 10^−5^	0.74	890	2.11 × 10^−6^	2600	1.84 × 10^−4^	8165	1.23 × 10^−5^	0.01	3.25	0.58	70.89
	24	2.02	6.52 × 10^−5^	0.76	818	4.00 × 10^−6^	3482	3.93 × 10^−4^	8651	1.24 × 10^−5^	0.03	3.66	0.44	74.52
200	0	1.56	6.16 × 10^−5^	0.87	445							15.36	0.59	56.36
	4	1.61	4.92 × 10^−5^	0.83	1262	1.86 × 10^−6^	2029	1.23 × 10^−4^	12,380	6.99 × 10^−6^	0.11	7.48	0.30	80.84
	8	1.71	4.63 × 10^−5^	0.89	1179	5.52 × 10^−4^	8917	3.55 × 10^−5^	10,600	4.55 × 10^−6^	2043	14.28	0.60	78.03
	24	1.56	4.84 × 10^−5^	0.89	769	4.19 × 10^−4^	11,240	1.95 × 10^−5^	15,960	6.03 × 10^−6^	2071	15.41	0.76	72.89
400	0	1.45	8.89 × 10^−5^	0.83	539							14.18	0.33	64.03
	4	2.11	5.70 × 10^−5^	0.82	1397	8.09 × 10^−7^	2182	1.12 × 10^−4^	12,240	7.97 × 10^−6^	0.10	8.19	1.11	82.69
	8	2.33	6.03 × 10^−5^	0.77	1079	2.09 × 10^−6^	1535	1.13 × 10^−4^	9707	1.24 × 10^−5^	0.01	4.49	0.61	76.00
	24	1.50	7.77 × 10^−5^	0.75	749	3.45 × 10^−6^	3506	3.50 × 10^−4^	11,970	1.05 × 10^−5^	0.01	3.77	0.31	72.16
600	0	1.40	1.22 × 10^−4^	0.82	490							19.02	0.85	60.42
	4	1.99	5.25 × 10^−5^	0.81	1350	1.93 × 10^−6^	1760	1.75 × 10^−4^	9684	1.19 × 10^−5^	0.17	6.52	0.57	82.09
	8	1.70	4.70 × 10^−5^	0.83	1026	3.30 × 10^−5^	15,580	2.63 × 10^−4^	15,860	5.32 × 10^−6^	0.45	6.72	0.45	74.76
	24	1.59	9.54 × 10^−5^	0.74	694	2.06 × 10^−6^	2402	3.15 × 10^−4^	6158	9.29 × 10^−6^	0.66	4.50	0.78	69.96

**Table 3:** Electrochemical parameters derived from polarization curves of mild steel in 1 M HCl solution at several concentrations of the PSL extract.

C_inh_ (ppm)	E_corr_ (mV)	b_a_ (mV/dec)	b_c_ (mV/dec)	i_corr_ (mA/cm^2^)	η (%)
0	−432.73	70.00	160.90	0.1271	
50	−441.14	76.90	159.90	0.0494	61.11
100	−447.77	96.50	140.10	0.0311	75.57
200	−456.77	102.60	142.20	0.0235	81.50
400	−448.47	112.10	124.90	0.0092	92.74
600	−464.32	98.60	114.10	0.0141	88.88

**Table 4:** Elemental analysis of steel sample specimens obtained from EDS spectra.

	Chemical Composition (w%)				
Sample	Fe	C	O	Cl	Mn
Air	95.52	3.35	0.23	--	0.9
0 ppm	60.98	4.22	29.65	4.62	0.51
400 ppm	85.16	5.02	8.90	0.19	0.71

**Table 5:** Thermodynamic adsorption parameters derived from Langmuir isotherm.

Test	K_ads_ (L/g)	ΔG_ads_ (kJ/mol)
PPC	42.55	−26.21
EIS	59.17	−27.22

**Table 6:** Frontier molecular orbitals (HOMO, LUMO) and electrostatic potential (ESP) of main compounds present in the PSL extract.

Compound	HOMO	LUMO	ESP
Phytol	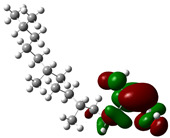	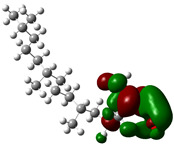	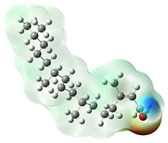
9,12,15-Octadecatrienoic acid, ethyl ester, (Z,Z,Z)-	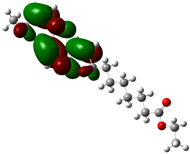	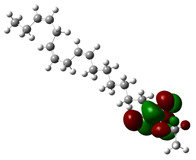	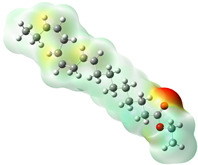
Phthalic acid, di(2-propylpentyl) ester	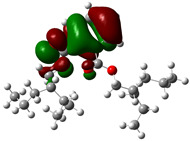	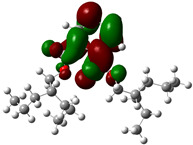	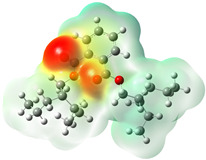
Linoleic acid ethyl ester	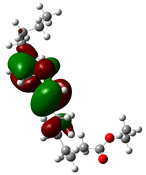	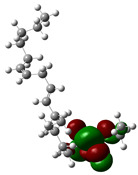	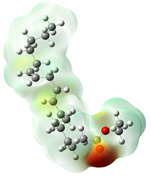

**Table 7:** Quantum chemical descriptors of the main compounds of the PSL extract.

Molecule	E_HOMO_ (eV)	E_LUMO_ (eV)	ΔE_gap_ (eV)	η_Q_ (eV)	χ (eV)	ΔN
Phytol	−6.379	0.206	6.585	3.220	3.014	0.619
9,12,15-Octadecatrienoic acid, ethyl ester, (Z,Z,Z)-	−6.009	0.495	6.504	3.252	2.757	0.652
Phthalic acid, di(2-propylpentyl) ester	−6.913	−1.422	5.491	2.727	4.149	0.523
Linoleic acid ethyl ester	−6.314	0.438	6.752	3.297	2.860	0.628

**Table 8:** Plant leaf extracts evaluated as eco-friendly corrosion inhibitors on mild steel in 1 M HCl solution.

Leaf Extract	C_inh_ (ppm)	Solution	Solvent	η (%)	Ref.
*Falcaria vulgaris*	800	HCl 0.5 M	Water	91 EIS	[63]
*Calamintha*	1500	HCl 1 M		87.57 EIS	[20]
*Saussurea obvallatta*	200	HCl 1 M	Methanol	90 EIS	[64]
*Tabebuia heterophylla*	440	HCl 1 M	Ethanol	94.8 WL	[36]
*Platanus acerifolia*	400	HCl 1 M	Ethanol/Water	91.5 PPC	[25]
*Mish Gush*	1200	HCl 1 M	Water	96 EIS	[34]
*Dolichandra unguis-cati*	760	HCl 1 M	Ethanol	93.6 WL *	[65]
*Pistia stratiotes*	400	HCl 1 M	Ethanol/Water	93.7 PPC	This study

* WL = weight loss.

## Data Availability

The data presented in this study are available on request from the corresponding author.

## Supplementary Materials

The following supporting information can be downloaded at: https://www.mdpi.com/article/10.3390/molecules29225243/s1, Figure S1: Examples of an EIS spectra fitting by using the electrical equivalent circuit. (a) 0 ppm at 4 h, (b) 600 ppm at 4 h; Table S1: Compounds identified from PSL extract using GC-MS analysis; Table S2: Condensed Fukui functions of the most reactive atoms of the chief constituents of PSL extract.
