# Supplementary material for: Insight into the Corrosion Inhibition Performance of Pistia stratiotes Leaf Extract as a Novel Eco-Friendly Corrosion Inhibitor for Mild Steel in 1 M HCl Solution
Source: Molecules. 2024 Nov 6;29(22):5243. doi: 10.3390/molecules29225243 (PMC11596646; doi:10.3390/molecules29225243)
Supplement: Supplementary file 1 [file molecules-29-05243-s001.zip › molecules-3277865-supplementary.pdf]

Table S1. Compounds identified from PSL extract using GC-MS Analysis.

	Compound	Rt (min)	Molecular weight	Molecular formula	Area (%)
1	Phytol	21.1	296	$C_{20}H_{40}O$	33.59
2	9,12,15- Octadecatrienoic acid, ethyl ester, (Z,Z,Z)-	21.57	306	$C_{20}H_{34}O_2$	20.98
3	Phthalic acid, di(2-propylpentyl) ester	25.89	390	$C_{24}H_{38}O_4$	14.03
4	Linoleic acid ethyl ester	21.50	308	$C_{20}H_{36}O_2$	11.15
5	Hexadecanoic acid, ethyl ester	19.91	284	$C_{18}H_{36}O_2$	9.60
6	Cyclotetrasiloxane, octamethyl-	6.86, 7.28	297	$C_8H_{24}O_4Si_4$	3.25
7	1,3-Dimethyl-1,3-bis(4-methylphenyl)-1,3-5disilacyclobutane	26.97	296	$C_{18}H_{24}Si_2$	3.22
8	Cyclooctasiloxane, hexadecamethyl-	16.49	593	$C_{16}H_{48}O_8Si_8$	2.37
9	Cycloheptasiloxane, tetradecamethyl-	14.47	519	$C_{14}H_{42}O_7Si_7$	1.80
10	Thieno[2,3-c]furan-3-carbonitrile, 2-amino-4,6-dihydro-4,4,6,6-tetramethyl-	6.57	222	$C_{11}H_{14}N_2OS$	--
11	1,4-Bis(trimethylsilyl)benzene	6.6	222	$C_{12}H_{22}Si_2$	--
12	Cyclotetrasiloxane, octamethyl-	6.86	297	$C_8H_{24}O_4Si_4$	--
13	α-N-Normethadol	13.29, 18.63, 19.3,	297	$C_{20}H_{27}NO$	--

14	Dihydroxanthin	19.48	308	$C_{17}H_{24}O_5$	--
15	Tricyclo[5.4.3.0(1,8)]tetradecan-6-one, 4-ethenyl-3-hydroxy-2,4,7,14-tetramethyl	19.72	305	$C_{20}H_{32}O_2$	--
16	Octasiloxane, 1,1,3,3,5,5,7,7,9,9,11,11,13,13,15,15-hexadecamethyl-	24.81	579	$C_{16}H_{50}O_7Si_8$	--

-- traces

Table S2. Condensed Fukui functions of the most reactive atoms of chief constituents of PSL extract.

Phytol			Octadecatrienoic acid, ethyl ester,			Phthalic acid, di(2-propylpentyl) ester			Linoleic acid ethyl ester		
A.	f_k^+	f_k^-	A.	f_k^+	f_k^-	A.	f_k^+	f_k^-	A.	f_k^+	f_k^-
C4	0.001	0.008	C1	0.058	0.015	C1	-0.003	0.036	C6	0.104	0.135
C17	0.188	0.160	C2	0.086	0.008	C2	-0.003	0.038	C7	0.075	0.072
C18	0.157	0.195	C11	0.139	-0.080	O3	0.033	-0.093	C9	0.051	0.104
C19	0.016	-0.039	O12	0.078	0.302	C4	0.098	0.115	C10	0.065	0.137
O20	0.045	0.087	O13	0.027	0.134	O5	0.103	-0.016	C18	0.155	0.006
H22	0.014	0.014	C16	0.032	0.004	C6	0.084	0.189	O19	0.093	0.108
H23	0.005	0.006	C17	0.022	0.014	C7	0.029	-0.065	O20	0.032	0.029
H24	0.008	0.005	C19	0.047	0.004	C8	0.060	0.209	H23	0.012	0.012
H25	0.012	0.008	C20	0.073	0.021	C9	0.124	0.205	H24	0.011	0.012
H26	0.006	0.006	H23	0.006	0.022	C11	0.089	0.224	H26	0.013	0.014
H27	0.019	0.013	H24	0.020	0.015	C12	0.019	0.130	H28	0.018	0.023
H29	0.010	0.011	H25	0.037	0.016	O13	0.065	-0.042	H30	0.026	0.022
H31	0.014	0.023	H26	0.037	0.016	C15	0.001	0.015	H31	0.011	0.014
H32	0.011	0.012	H27	0.015	0.014	C16	-0.004	0.037	H32	0.010	0.010
H33	0.004	0.007	H28	0.015	0.014	C17	-0.003	0.037	H33	0.033	0.038
H34	0.005	0.023	H39	0.036	0.038	H24	0.036	0.044	H34	0.026	0.026
H35	0.007	0.020	H40	0.036	0.038	H25	0.042	0.034	H35	0.024	0.022
H37	0.019	0.011	H41	0.014	0.018	H26	0.044	0.036	H36	0.026	0.062
H41	0.013	0.014	H42	0.014	0.018	H27	0.036	0.042	H37	0.030	0.038
H42	0.009	0.010	H43	0.014	0.020	C35	-0.001	0.029	H38	0.028	0.030

H44	0.011	0.016	H44	0.014	0.020	H39	0.018	0.001	H39	0.008	0.011
H46	0.014	0.013	H45	0.022	0.014	C41	-0.004	0.026	H40	0.025	0.042
H47	0.021	0.012	H46	0.014	0.020	C44	-0.002	0.026	H41	0.014	0.020
H50	0.024	0.029	H47	0.038	0.016	C47	-0.004	0.023	H42	0.013	0.022
H51	0.009	0.008	H48	0.038	0.016	C50	-0.003	0.027	H45	0.012	0.012
H52	0.044	0.032	H49	0.022	0.020	C53	0.000	0.030	H47	0.012	0.014
H53	0.036	0.023	H50	0.024	0.038	C56	-0.005	0.069	H49	0.017	0.010
H54	0.043	0.036	H51	0.022	0.019	C59	0.000	0.071	H50	0.021	0.014
H55	0.042	0.030	H52	0.022	0.019	C62	0.001	0.031	H51	0.011	0.009
H56	0.036	0.045	H53	0.015	0.012				H52	0.029	0.009
H57	0.054	0.042							H53	0.054	0.020
H58	0.045	0.019							H54	0.023	0.015
H59	0.044	0.044							H55	0.012	0.006
H60	0.027	0.024							H57	0.025	0.015
H61	0.055	0.049									

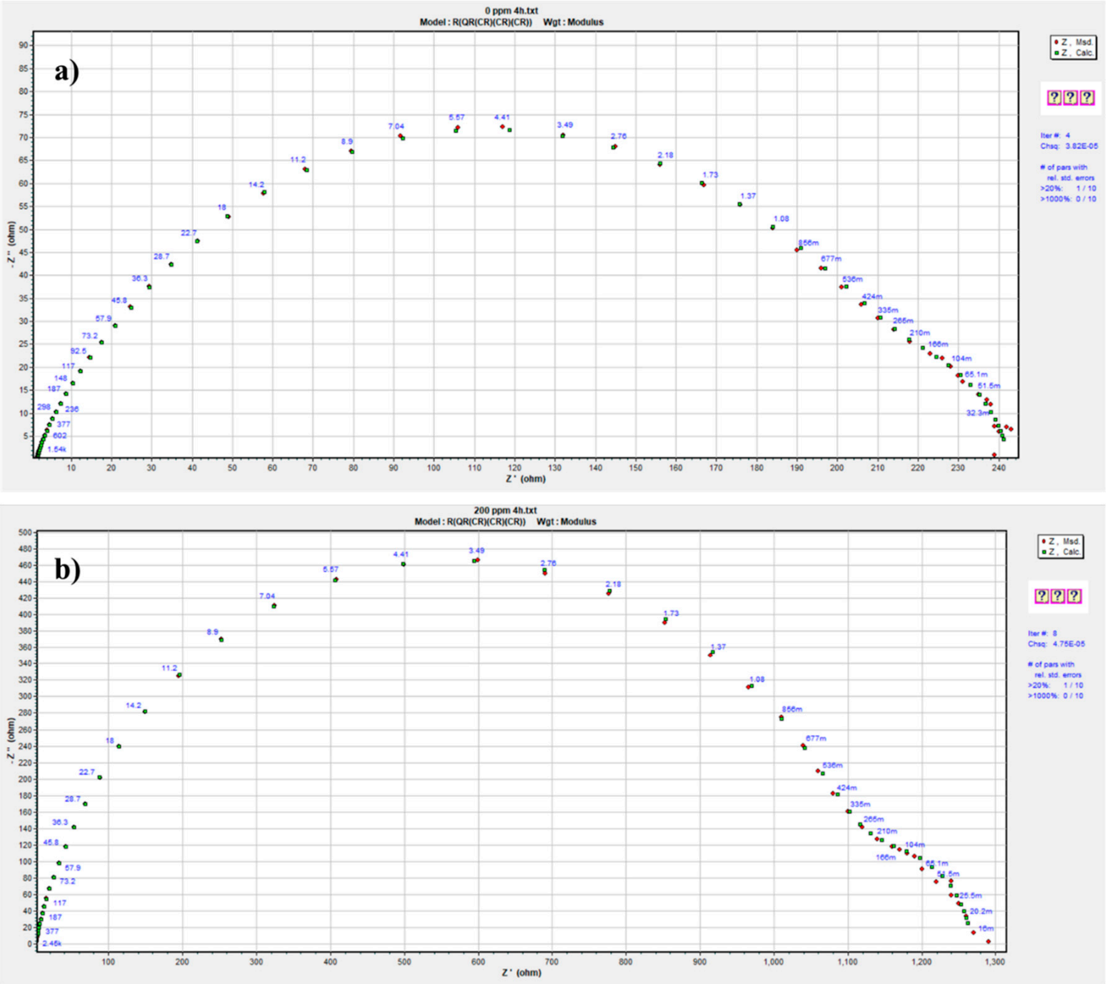

Figure S1. Examples of an EIS spectra fitting by using the electrical equivalent circuit. a) 0 ppm at 4h, b) 600 ppm 4h.
